# Factors affecting COVID-19 outcomes in patients with congenital heart disease

**DOI:** 10.1017/S1047951120004825

**Published:** 2020-12-17

**Authors:** Aisha Ahmad, Marcellina B. A. Chiejina, Shifa Bangi

**Affiliations:** Imperial College London, Faculty of Medicine, School of Medicine, London, UK

**Keywords:** Coronavirus, congenital heart disease, COVID-19, outcomes

Haiduc et al attempt to summarise the current literature surrounding the effect of congenital heart disease (CHD) on COVID-19 outcomes.^1^ We propose further consideration of the following factors: age, type and severity of CHD, and comorbidities.

This review directly compares studies and case reports of patients with ages ranging from 3 months to 76 years. Discrepancy in ages is important to consider since COVID-19 is known to affect infants less than 1 year of age more severely.^2^ This may be due to low T-cell activation, reduced expression of ACE2 in the lungs and other developmental differences.^2^ Older patients are likely to have developed more comorbidities which may be the reason for the presentation of worse COVID-19 symptoms, rather than being due to their CHD alone. Literature also shows racial disparities in the severity of COVID-19 which may confound the effect of CHD on outcomes.^3^

Contrary to this review, there is evidence that individual defects such as single ventricular defects are not sufficient cause alone for worse COVID-19 outcomes.^4^ Lewis et al reported that out of 53 CHD patients, there appeared to be no correlation between the complexity of the CHD and the subsequent infection-related cardiac decompensation displayed by the patient.^4^ This may explain why patients with similar CHDs experience different disease trajectories. Therefore, using only the CHD status is not a sufficient indicator for poor outcomes and markers of symptom progression such as raised cardiac troponins upon admission may prove more useful.^5^ This is particularly relevant since CHD patients with infection-induced myocardial injury are at a higher risk of complications.^5^ Treatment protocols used to manage infections should also be considered amongst the studies included in the review. For example, Non-steroidal anti-inflammatory drugs use in early infection is thought to be controversial due to reports of causing more complications when administered for respiratory tract infections.^6^

From the studies included in the review, 24% of the patients had comorbidities with the most frequent being genetic conditions, type 2 diabetes mellitus, and chronic kidney disease.^1^ Massin et al demonstrates that a significant proportion of children with CHD have associated non-cardiac comorbidities including genetic conditions, with the most common being trisomy 18.^7^ These comorbidities make it difficult to assess the direct effect of CHDs on outcomes. Similarly, Agarwal et al show that adults with CHD are twice as likely to have non-cardiac comorbidities compared to their non-CHD counterparts.^8^ Additionally, for patients below the age of 40, the more severe the CHD, the higher the risk of developing non-cardiac comorbidities.^8^

In conclusion, in order to determine causality or a significant correlation between COVID-19 prognosis and CHD, this review needs to stratify CHD according to type of defect and severity, whilst controlling for confounders such as age and comorbidities.
